# Supplementary material for: Understanding ultrafast free-rising bubble capturing on nano/micro-structured super-aerophilic surfaces
Source: Nat Commun. 2025 Apr 17;16:3682. doi: 10.1038/s41467-025-59049-x (PMC12006317; doi:10.1038/s41467-025-59049-x)
Supplement: Supplementary file 1 — Supplementary Information [file 41467_2025_59049_MOESM1_ESM.pdf]

Supplementary Information for:

Understanding ultrafast free-rising bubble capturing on nano/micro-structured super-aerophilic surfaces

Yue Hu[1], Zhenbo Xu[2], Haotian Shi[1], Benlong Wang[1,3], Liqiu Wang[4]*, Lu-Wen Zhang[1]*

[1]Department of Engineering Mechanics, School of Ocean and Civil Engineering, Shanghai Jiao Tong University; Shanghai, 200240, China.

[2]Department of Architecture and Civil Engineering, City University of Hong Kong; Hong Kong, 999077, China.

[3]Key Laboratory of Hydrodynamics (Ministry of Education), School of Ocean and Civil Engineering, Shanghai Jiao Tong University, Shanghai 200240, China

[4]Department of Mechanical Engineering, The Hong Kong Polytechnic University, Hong Kong, 999077, China.

*Corresponding author:

Liqiu Wang (email: liqiu.wang@polyu.edu.hk)

Lu-Wen Zhang (email: lwzhang@sjtu.edu.cn)

This file includes:
Supplementary Figures 1–27
Supplementary Tables 1–2
Supplementary Notes 1–5
Supplementary References 1–16

Supplementary Figures 1–27

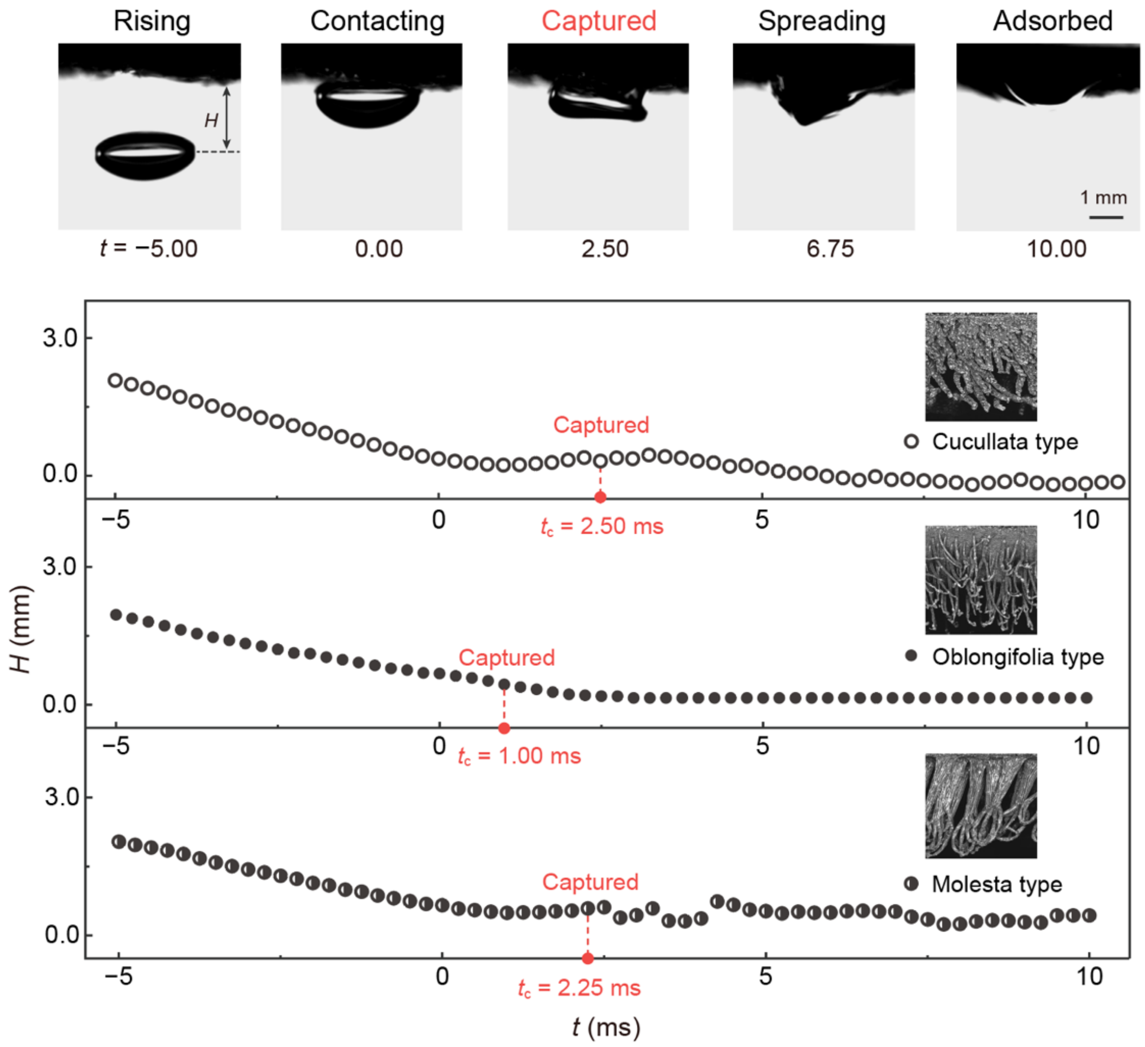

Supplementary Fig. 1. Ultrafast bubble capture on the Salvinia leaves. The high-speed image sequence shows a bubble with D_0 = 2.4 mm contacts the Salvinia leaf. Dots display the centroid position *H* of the bubble as a function of time *t*. Bubble capture occurs during the first contacting with Salvinia leaves, despite the varying species, with all capture time below 2.5 ms.

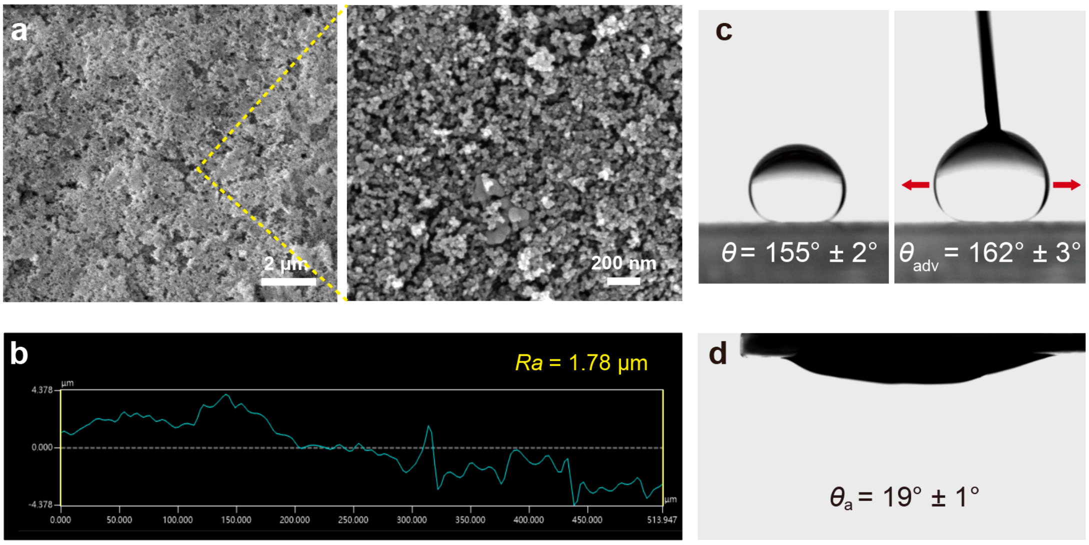

Supplementary Fig. 2. Characterizations of the aerophilic surfaces. a SEM images of the aerophilic surfaces after decorating the samples with silanized silica nanoparticles. **b** The texture profile of the surface in **a** measured by an upright laser confocal microscope showing the arithmetic average roughness *Ra* = 1.78 μm. **c** A sessile droplet with a volume of ~10 μL on the FH surface with an apparent contact angle θ of 155° ± 2°. The advancing angle θ_{adv} is measured before the contact line moves during increasing the drop volume, displaying the θ_{adv} of 162° ± 3°. **d** The image of a bubble on FH surface shows bubble contact angle θ_a of 19° ± 1°.

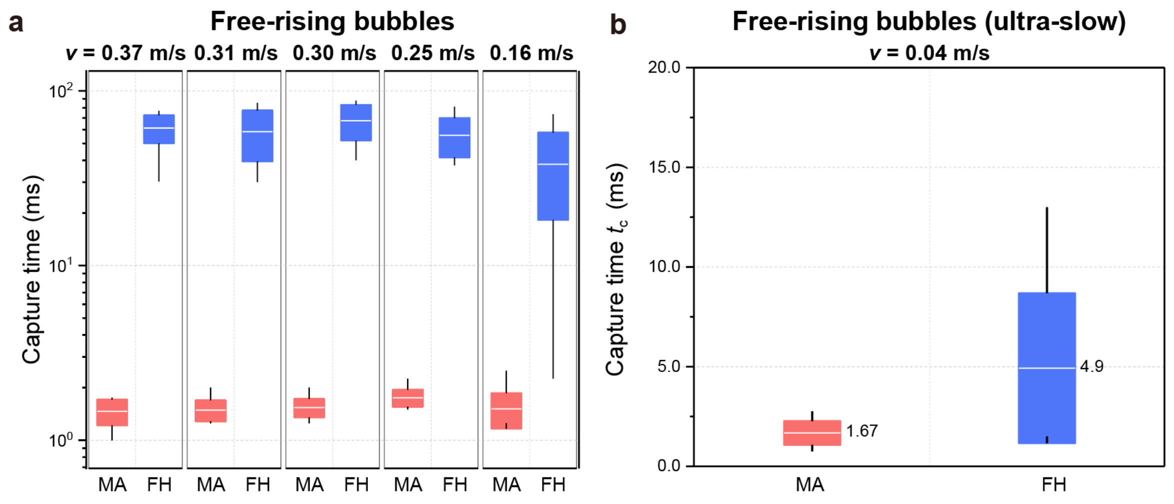

Supplementary Fig. 3. Capture time of free-rising bubbles on MA and FH surface at varying releasing distances. a Releasing distance includes 60mm, 20 mm, 15 mm, 10 mm, and 5 mm, resulting in approach velocities of 0.37, 0.31, 0.30, 0.25, 0.16 m/s. While immediate bubble capture occurs on MA surface with average capture times in a range of 1.46 to 1.75 ms, bubble bounce is observed on FH surfaces in these cases. **b** Capture time of free-rising bubbles at a specific low approach velocity of 0.04 m/s with releasing distance of ~1.74 mm. Immediate bubble capture is observed on MA surface with average capture time of 1.67 ms and small standard deviation. No bubble bounce occurs on FH surfaces with an average capture time of 4.9 ms and large standard deviation. The black vertical lines on the bars indicate the maximum and minimum values of the dataset.

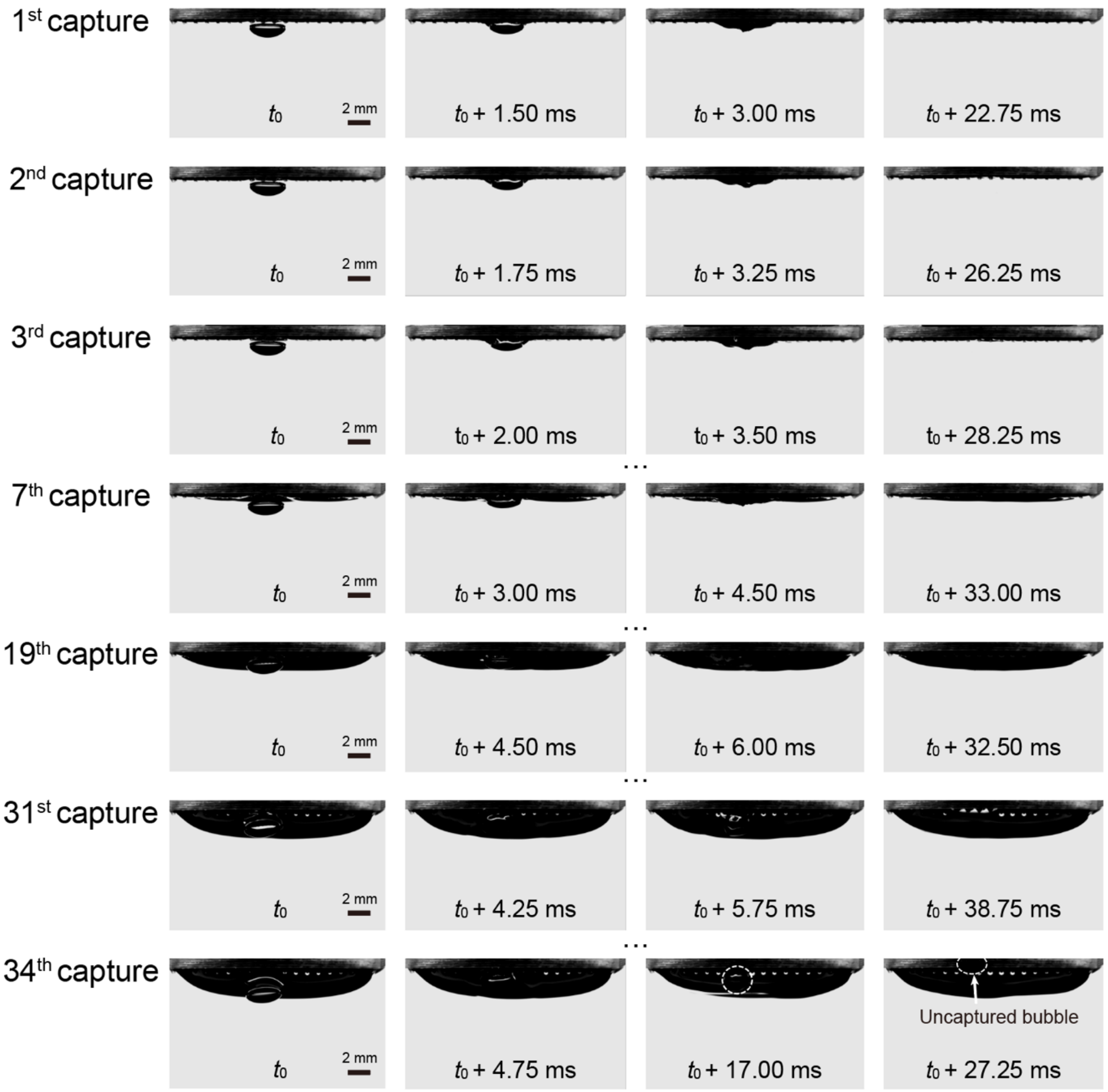

Supplementary Fig. 4. Continuous bubble capture on the MA surface. The experiment was repeated 34 times until the last time the bubble could not be captured and slid away from the surface.

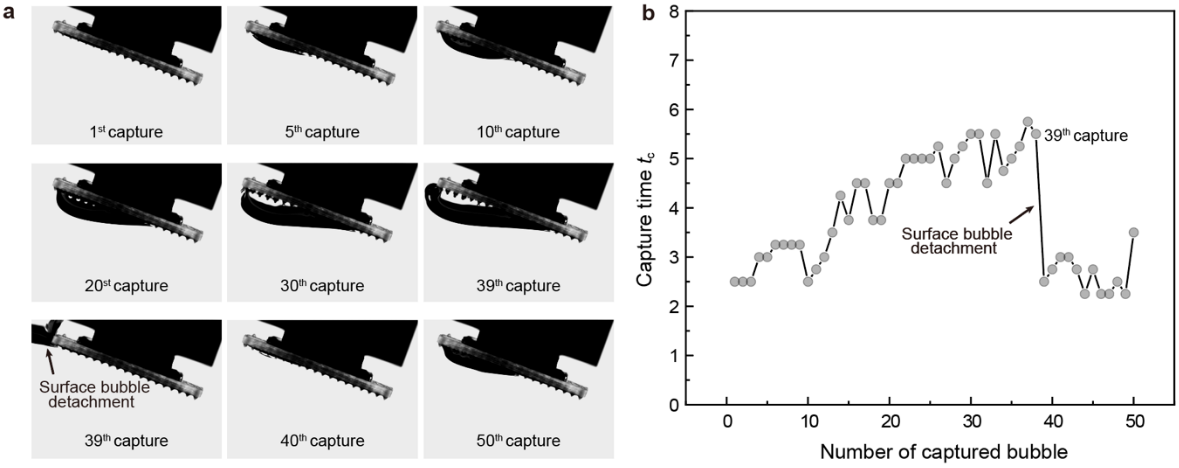

Supplementary Fig. 5. Continuous bubble capture on the tilted MA surface. (a) The image sequence of 50 consecutive bubble capture experiments. The MA surface was inclined at an angle of 20°. (b) The capture time as a function of the number of bubbles captured.

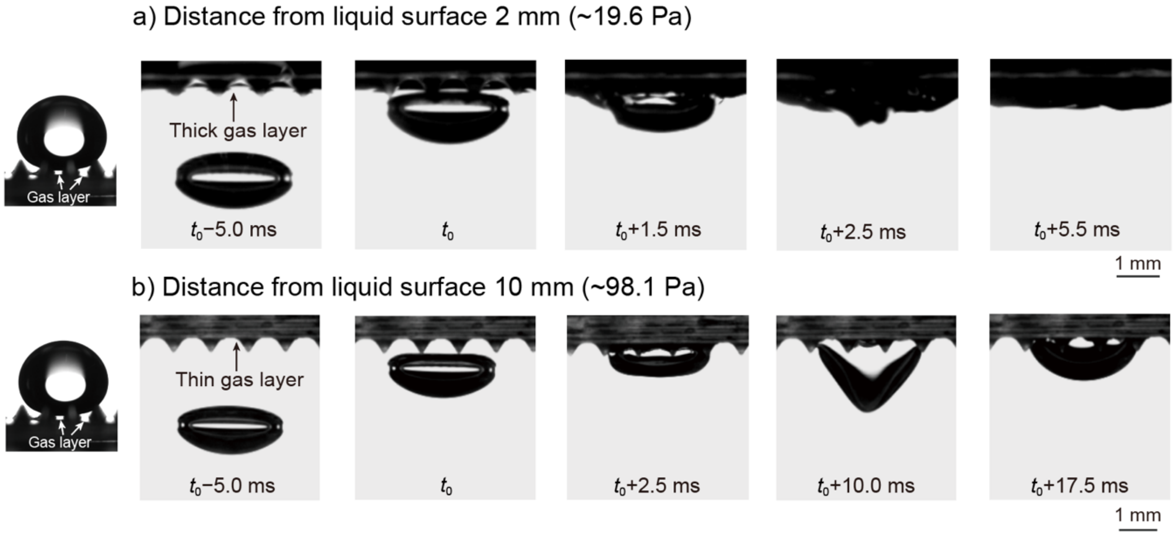

Supplementary Fig. 6. The high-speed image sequences of bubble behaviors on the super-aerophilic silanized microstructured surfaces without nanostructures. The surface distance from the liquid is tested at two values: a) 2 mm, corresponding to a hydrostatic pressure of 19.6 Pa, and b) 10 mm, corresponding to a hydrostatic pressure of 98.1 Pa. The substrate was first treated for plasma treatment (250 W, 180 s) to obtain surface-active hydroxyl groups after clean and dry, followed by a silanization reaction for 2 hours under the conditions of a silane concentration of 5.0×10^{-3} M, a temperature of 110°C, and a reduced pressure of 0.2 atm. When microstructures are not coated with nanoparticles but are instead functionalized with silane to create a chemically super-aerophilic surface, the bubble can also rupture rapidly (t_c = 2.1 ms ± 0.6 ms under 19.6 Pa and t_c = 2.6 ms ± 0.3 ms under 98.1 Pa), with only a small difference compared to the nanoparticle coated microstructure (t_c = 1.5 ms ± 0.1 ms). Although the plastron formed on the silanized surface appears to be metastable underwater and tends to be easily lost under hydrostatic pressure or long-time water immersion, leaving only a thin gas layer insufficient for complete bubble absorption, it is still sufficient for quick coalescence.

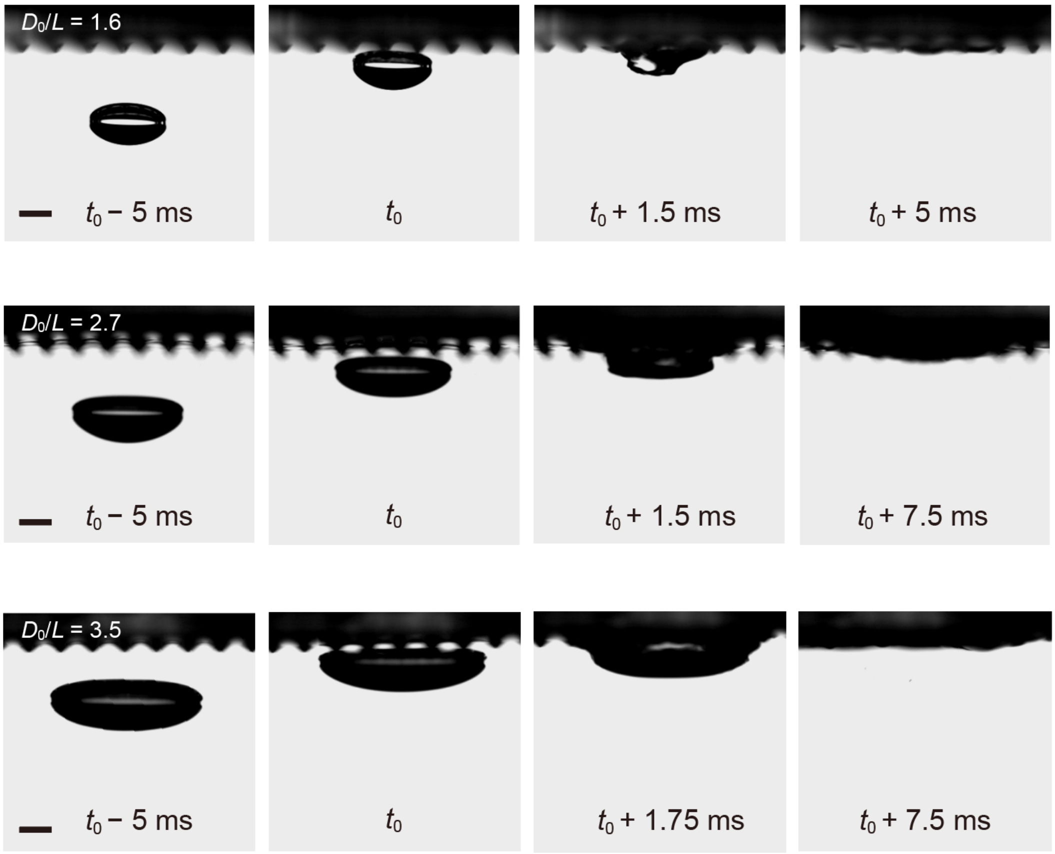

Supplementary Fig. 7. Bubble capture dynamics with varying D_0/L. High-speed image sequences of bubble dynamics on the MA surface with D_0/L = 1.6, 2.7 and 3.5. All cases exhibit bubble capture when the first contact with the surfaces. Scalar bars represent 1 mm.

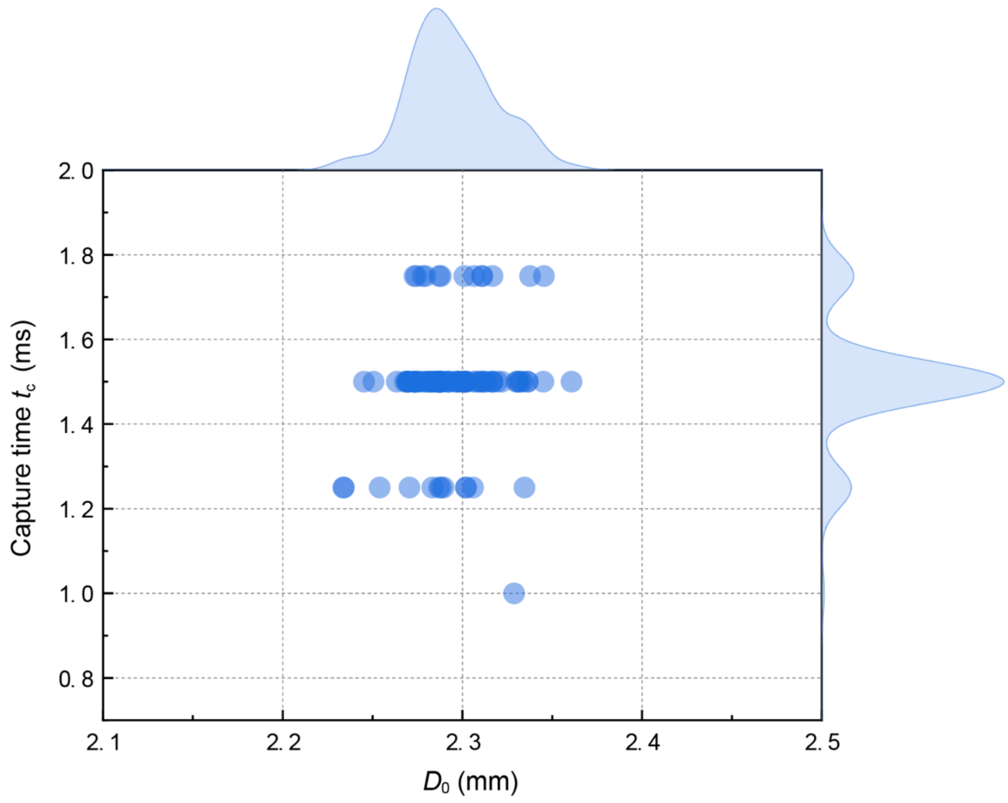

Supplementary Fig. 8. Statistical capture time tests of 100-repetitive bubble capture events on MA surface. The marginal layers show distribution curves of bubble diameter D_0 and capture time. The microcone array parameters on the MA sample surface are b/a = 1.7, L = 1 mm. The bubble released distance is 20 mm.

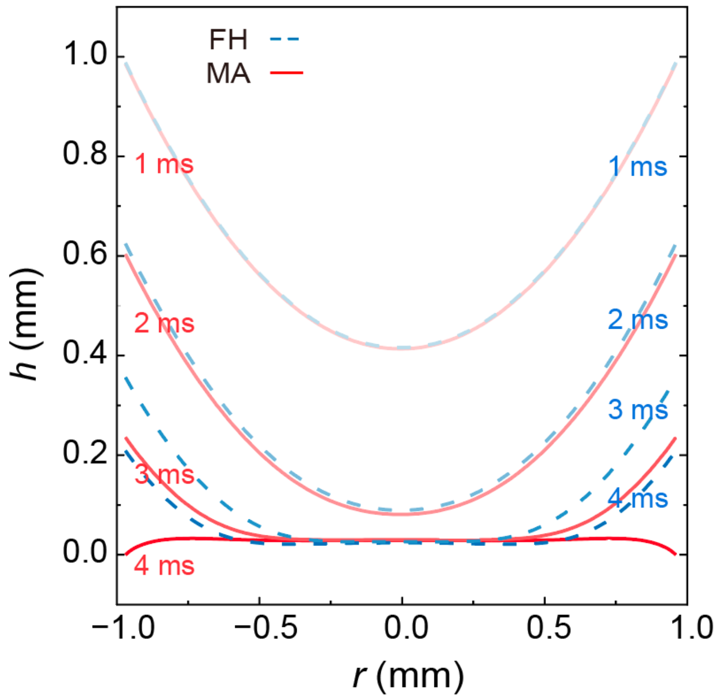

Supplementary Fig. 9. The spatio-temporal evolutions of the film thickness on the MA and FH surface. The evolutions of film thickness h in a 4-ms interval prior to contacting surfaces are evaluated by our theoretical model for the cases shown in Fig. 2d. At the same moment, h on the MA surface is always smaller than that on the FH surface, and attenuates faster during the bubble rising process, which proves the enhanced film drainage under the pinning state of three-phase contact lines.

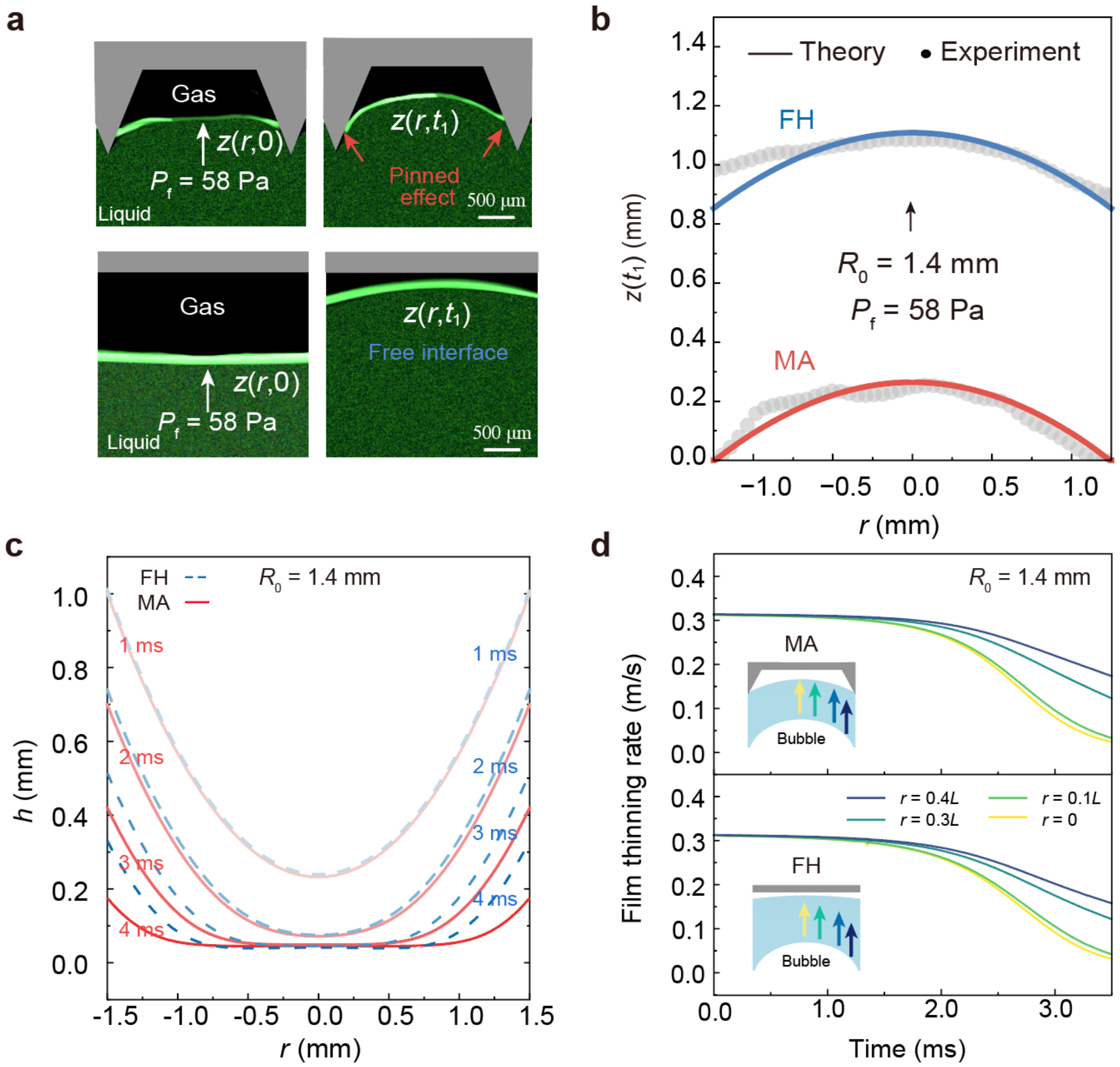

Supplementary Fig. 10. The theoretical estimation of LGI profiles and drainage rate driven by a rising bubble with D_0 = 2.8 mm. a The fluorescent reflection images of the LGI motion during the bubble approaching MA and FH surfaces, with averaged film pressure of approximately 58 Pa. The triangular ridge protrusions with a conical spacing L of 2.5 mm, a base radius a of 0.34 mm, and a height b of 1.2 mm. **b** The ultimate interface profiles $z(t_1)$ exhibit high consistency with the experimental results extracted from fluorescence reflection images in **a**. **c** The spatial-temporal evolutions of the film thickness on the MA and FH surface. **d** The film thinning rate predicted by our model at locations $r = 0{-}0.4L$. The accelerated film drainage is also observed on the MA surface, but the increment of the rate moderates due to the decreased film pressure than that formed by a rising bubble with D_0 = 1.6 mm.

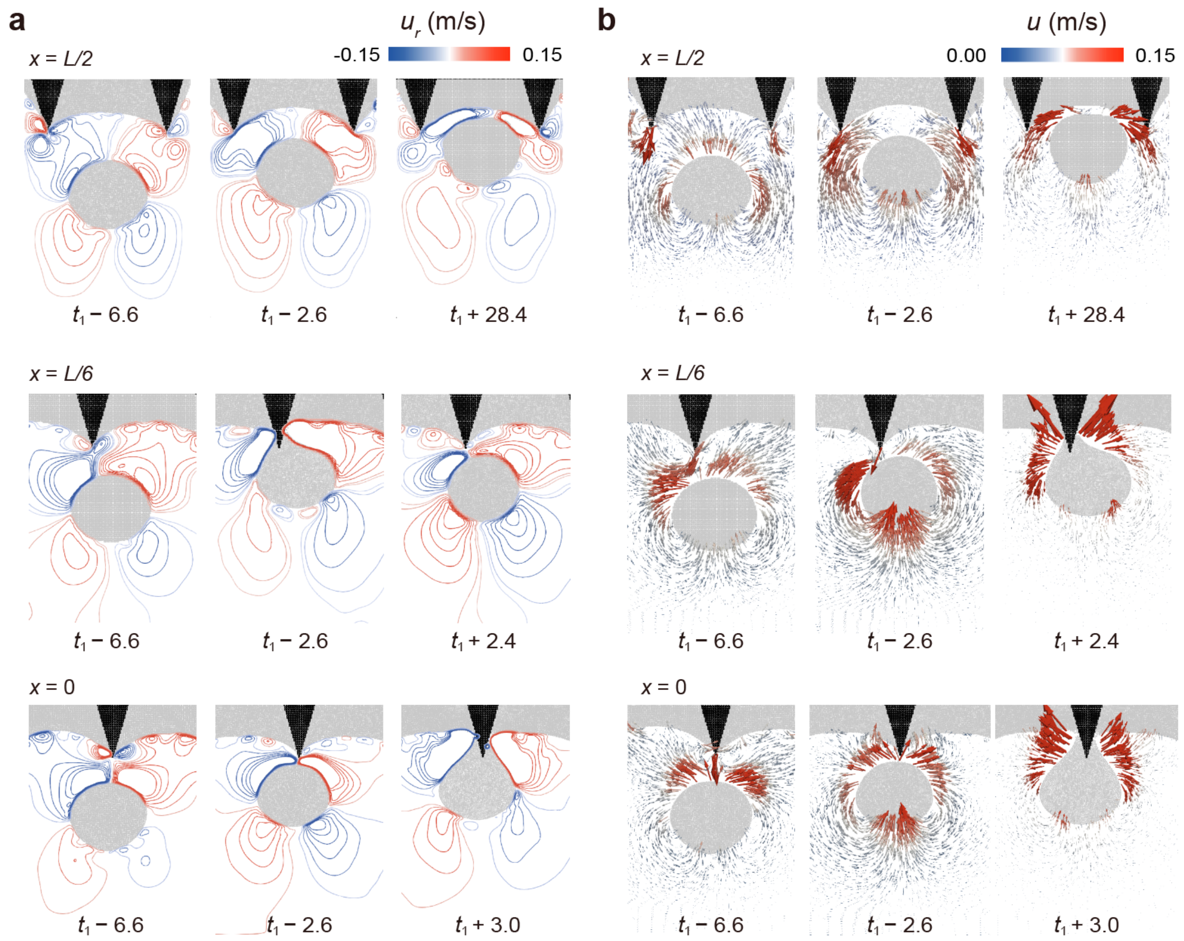

Supplementary Fig. 11. Drainage velocity field at three distances to the conical tip. Three distances x are $L/2$, $L/6$, and 0, respectively, and all results are obtained by SPH simulations. **a**, Horizontal velocities u_r contour maps and **b**, velocity u vector fields show accelerated fluid flow in the liquid film when bubble released closer to TPLs.

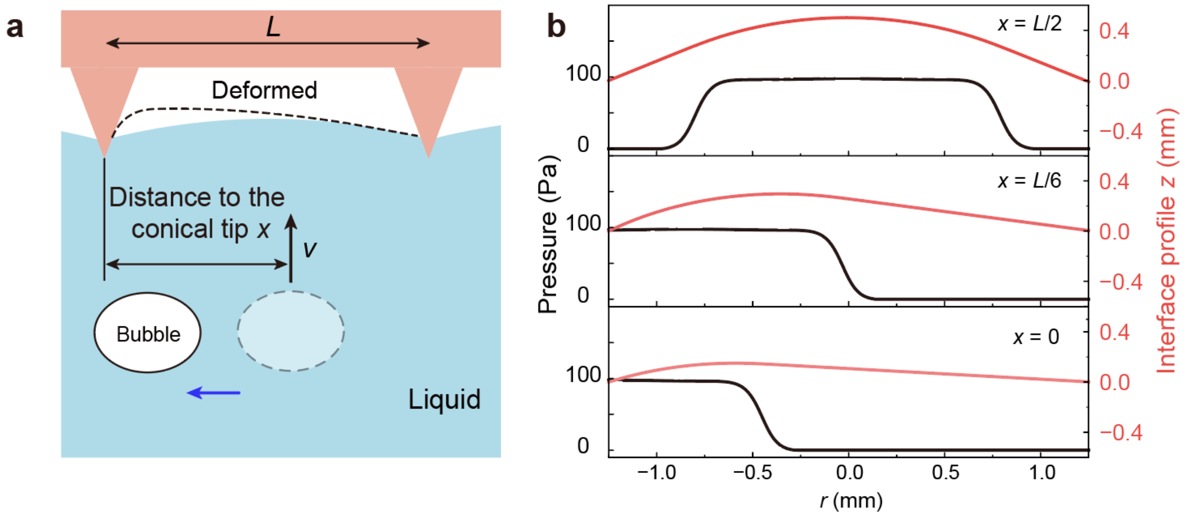

Supplementary Fig. 12. Liquid-gas interface deformation estimation at three locations on the MA surface. a Illustration of bubbles released at varying locations within two conical tips, and x represents the distance between the bubble centroid and the conical tip. **b** Distribution of the film pressure load applied, and **c**, ultimate interface profiles of LGI at $x = L/2$, $L/6$, and 0 (L = 2.5 mm). The interface profiles z are predicted by Young-Laplace equation $z''/2[1+(z')^2]^{3/2} + P_f(r)\gamma = 0$, where $P_f(r)$ is evaluated by Eq. S3. The boundary condition is set as $z(r = \pm L/2) = 0$. The results demonstrate that the pinning three-phase contact lines restrict the uplift displacement of liquid-gas interfaces, thus benefiting the fast film thinning process.

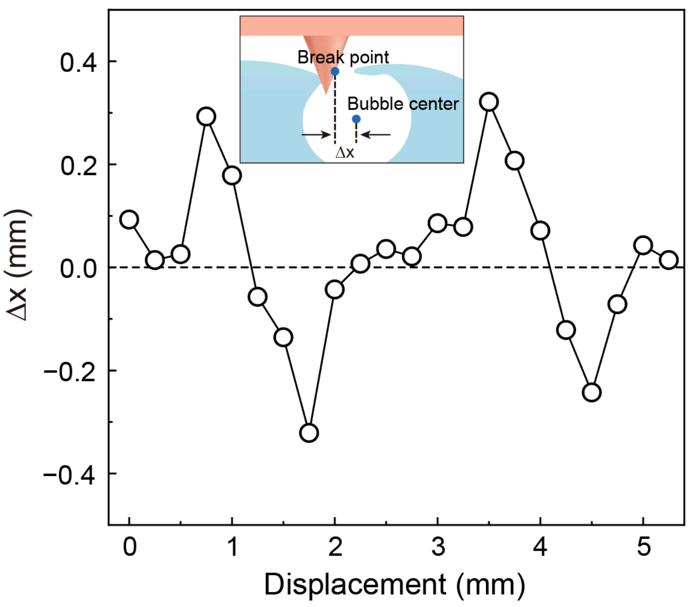

Supplementary Fig. 13. The distance Δ*x* between the bubble break point and the bubble centroid on several contact locations with a horizontal increment of 200 µm. The film breakpoint is the location where the contact angle of the bubble displays abrupt change. Δ*x* is measured to pinpoint bubble rupture locations, and its absolute value increases dramatically at the same positions where the capture time reaches ~1.5 ms, indicating the gas bridge forms at the position near the three-phase contact lines. The displacement = 0 corresponds to the tip of the first protrusion.

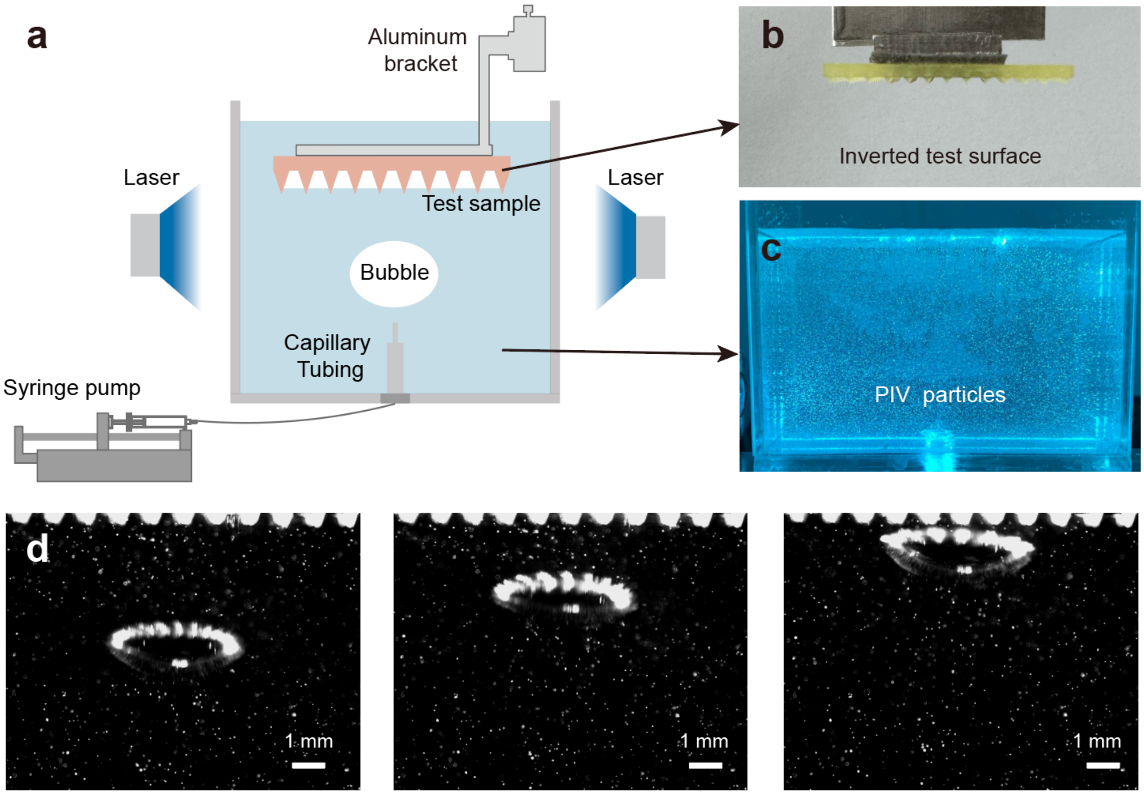

Supplementary Fig. 14. Device setup for the PIV experiment. a Experimental system for PIV tests. **b** An optical image of a testing sample invertedly settled underwater. **c** The PIV particles under laser sheet formed by two laser devices. **d** The original PIV images of a bubble with D_0/L= 3.0 approaching the MA surface with b/a = 3.5, recorded by a high-speed camera with 4000 fps. The time interval is 5 ms.

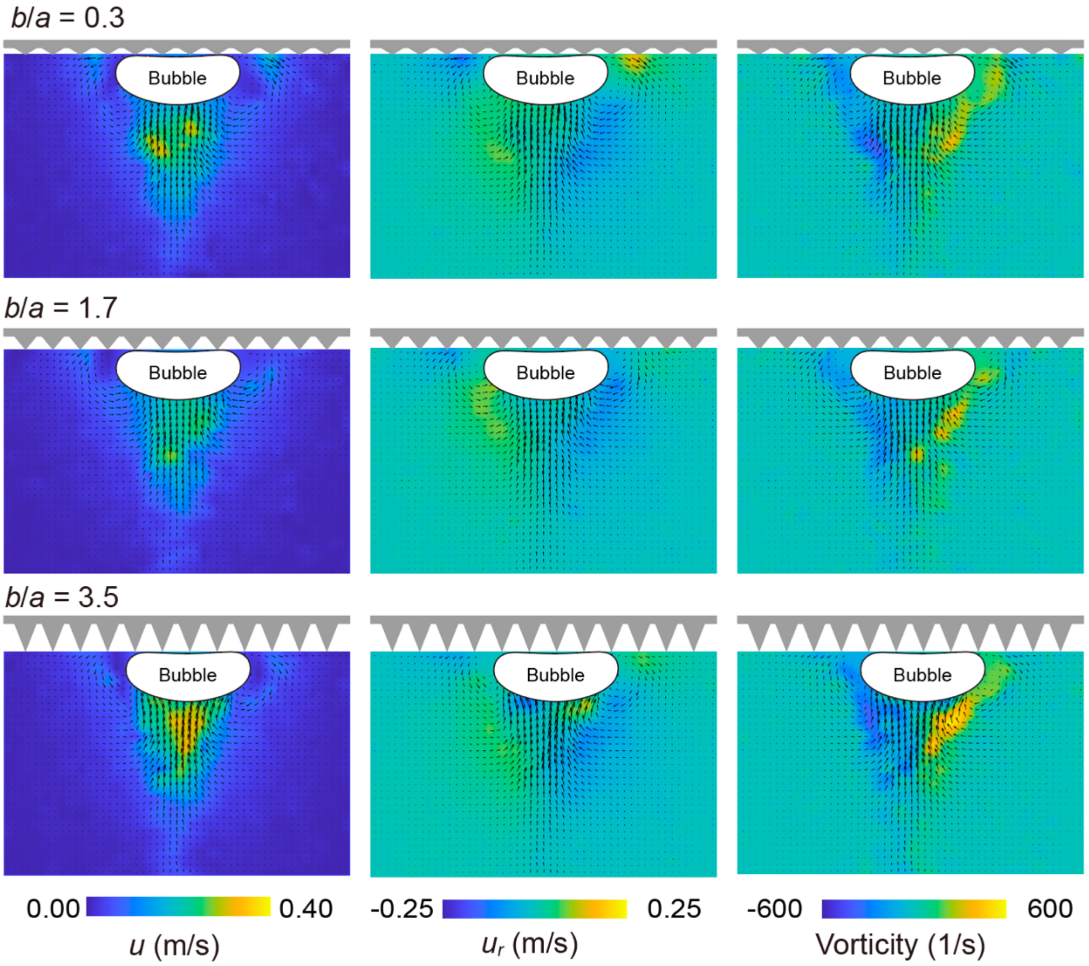

Supplementary Fig. 15. The velocity and vorticity maps on MA surfaces with varying geometrical parameters *b*/*a*. By employing the PIV tests, we obtain the distributions of velocity u, the horizontal velocity u_r, and the vorticity colored in magnitude when bubbles contact the MA surfaces with an increasing b/a from 0.3 to 3.5. The growth b/a expands the thickness of the gas layer, leading to larger liquid velocity and vorticity on the MA surface with greater b/a.

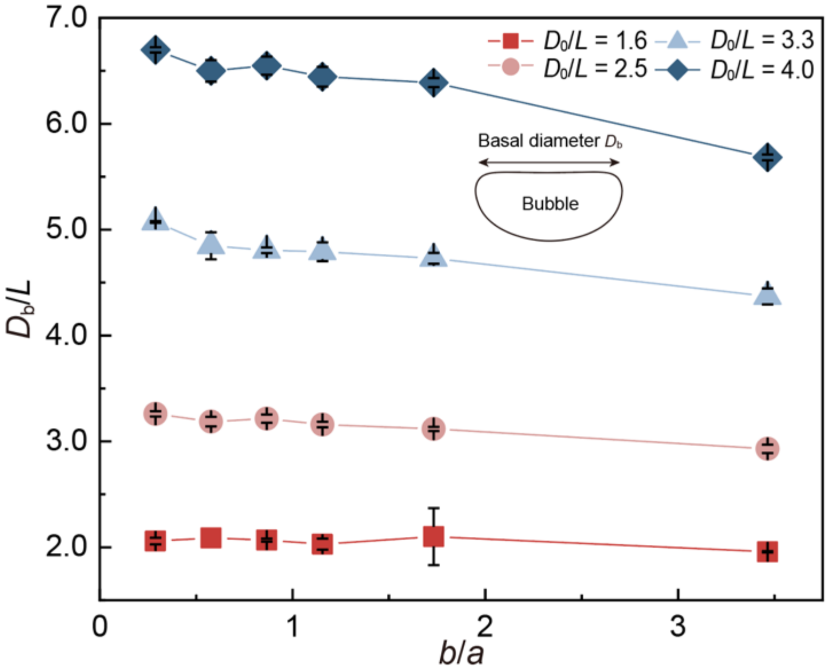

Supplementary Fig. 16. The dimensionless basal diameter of bubble D_b/L changes with b/a. The dimensionless basal diameter D_b/L declines as the b/a ratio increases, a trend observable across bubbles of varying sizes, suggesting that the bubble exhibits reduced deformation and forms a smaller liquid film area between the MA surface and the bubble. Error bars represent standard deviation calculated from three independent experiments.

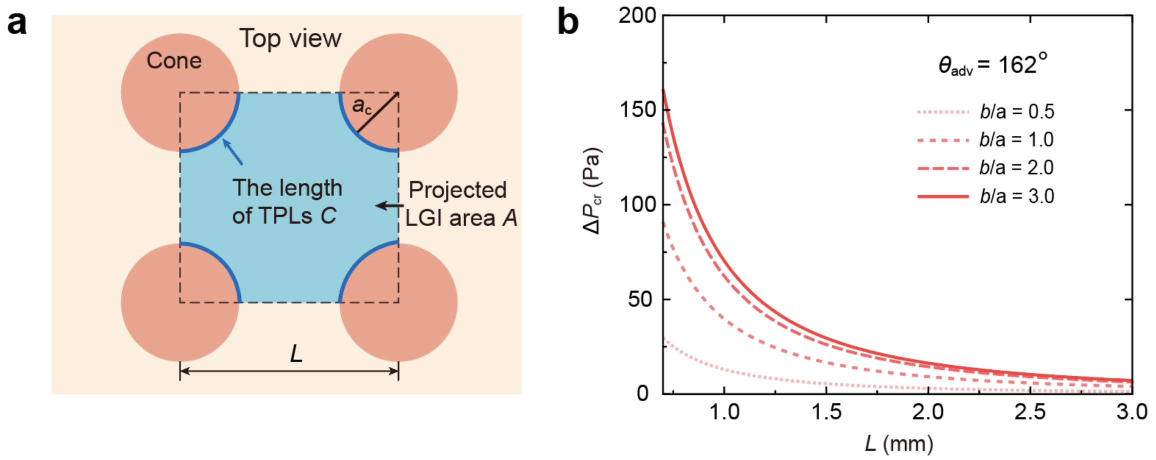

Supplementary Fig. 17. The critical threshold ΔP_{cr} evaluation on the MA surface. a Top view schematic of the geometry of a meniscus among a unit of four cones on the MA surface. **b** The relationship of the conical distance L and the ΔP_{cr} in the cases of b/a = 0.5, 1.0, 2.0, and 3.0, predicted by Eq. S12.

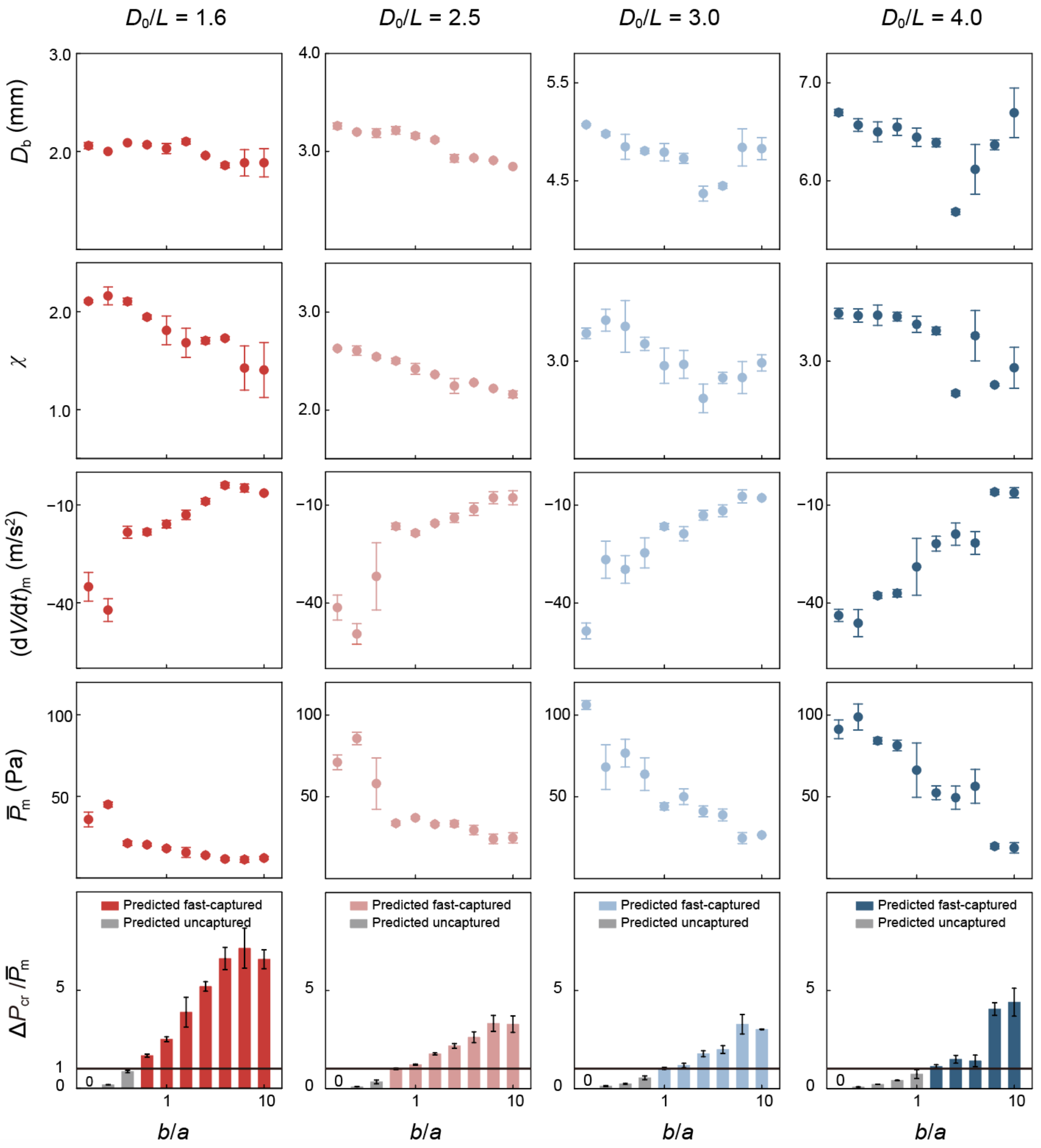

Supplementary Fig. 18. The data for the predictions of the fast-captured phenomenon on MA surface. The maximum deceleration $(dV/dt)_m$, and the contemporaneous χ and the bubble basal diameter D_b are obtained from the experiments. The maximum average film pressure $\bar{P}_m$ during the bubble contact is calculated by Eq. S4. For the cases of $\Delta P_{cr}/\bar{P}_m \geq 1$, the bubble is predicted uncaptured at the first contact, otherwise the bubble can be fast captured by the MA surface. Error bars represent standard deviation of three independent experiments.

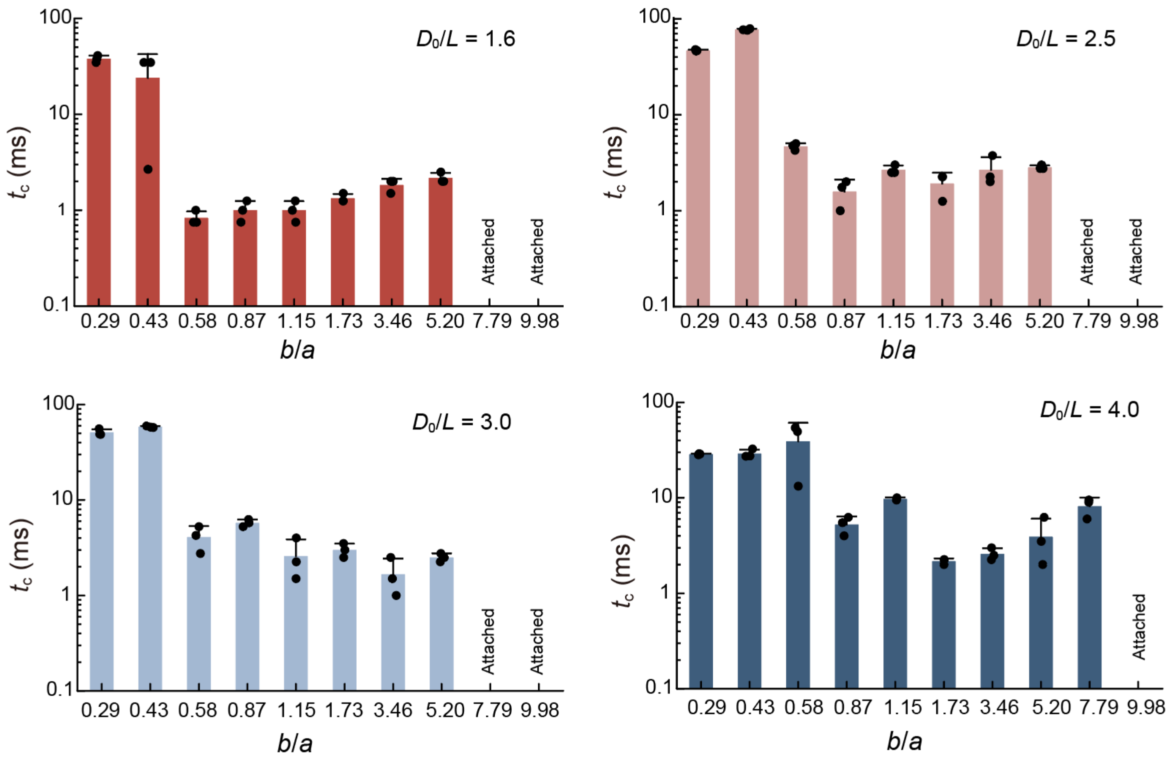

Supplementary Fig. 19. The capture time t_c of different dimensionless bubble sizes D_0/L on the MA surfaces with varying b/a. For bubbles unable to be captured fast, the t_c is relatively high, and reaches tens of milliseconds, while the fast captured cases show t_c below 10 ms. When b/a exceeds ~7.8, bubbles will suspend over the microstructures and fail to be captured. Error bars represent standard deviation of three independent experiments.

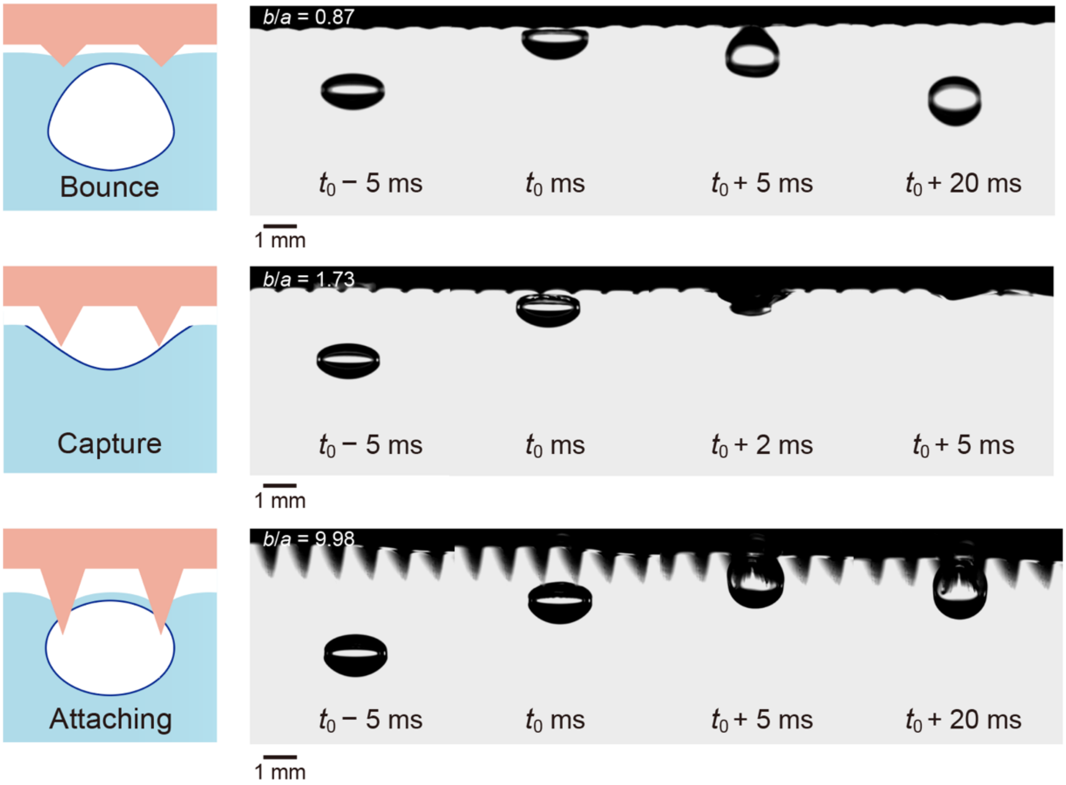

Supplementary Fig. 20. Three distinct bubble behaviors on the MA surface. High-speed camera photograph sequences illustrate the distinct behaviors of bubbles with D_0 = 1.6 mm upon initial surface contact. For b/a = 1.73, the bubble is rapidly captured, while for b/a = 0.87, it exhibits bouncing behavior, and for b/a = 9.98, it becomes pinned on the structures.

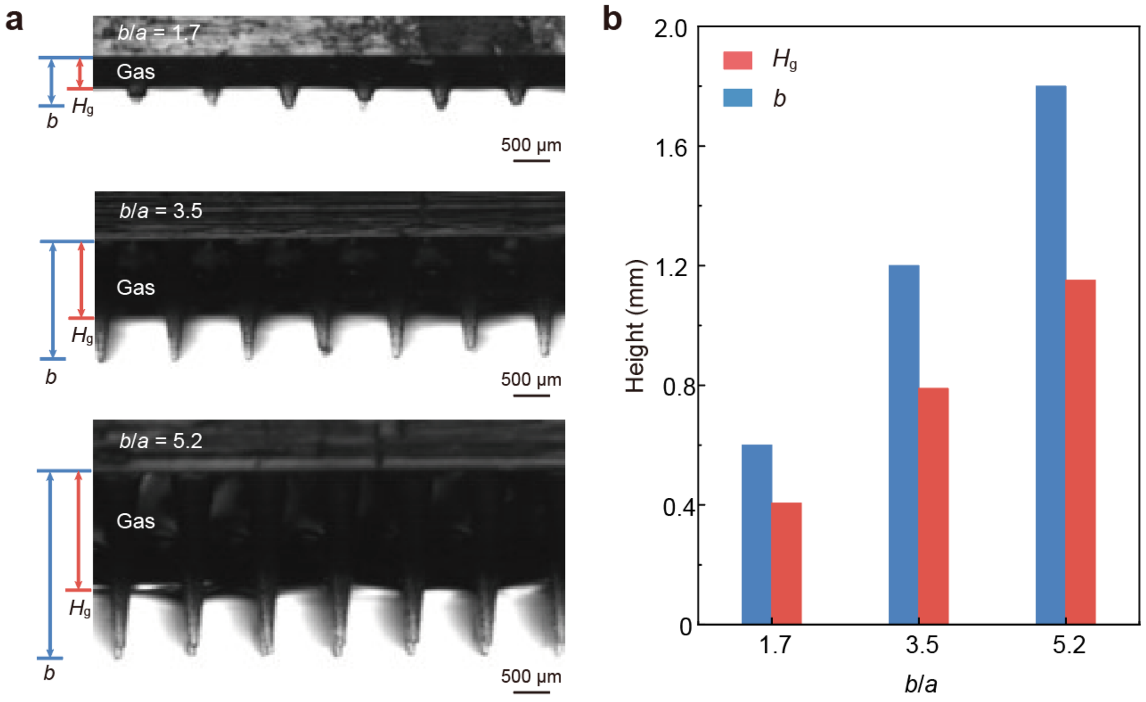

Supplementary Fig. 21. The height of gas layer H_g on the MA surface with varying *b*. a Optical images of the gas layer when the MA surfaces with *b*/a = 1.7, 3.5, and 5.2 immersed underwater. **b** The height of cones without an encapsulated gas layer increases with the growth of *b*/a.

a

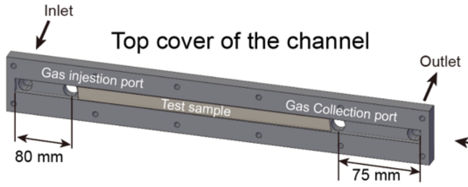

b

Sectional area (mm^2)	Flow velocity (m/s)	Reynolds number Re
60	0.28	2083
60	0.33	2500
60	0.39	2917
60	0.44	3333
60	0.50	3750
60	0.61	4583
60	0.72	5417

c

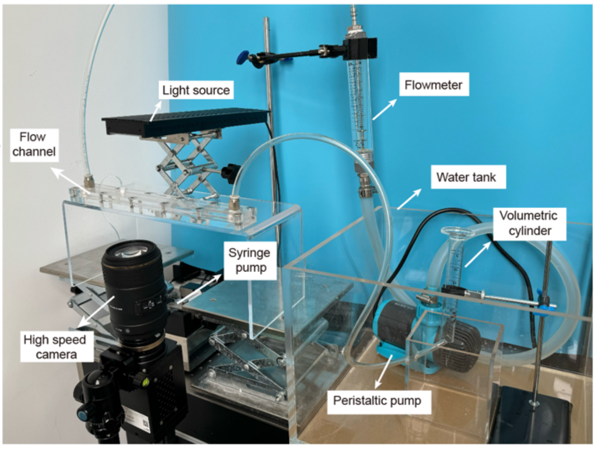

Supplementary Fig. 22. The experimental setup of the bubble capture system in a flow environment. a The top cover model of the channel embedded the gas injection port and gas collection port. **b** The Reynolds numbers and corresponding velocities and sectional area used in the experiments. **c** The setup of the entire system consists of a flow channel, a submerged pump, a syringe pump, a bubble collection section, and a high-speed camera system.

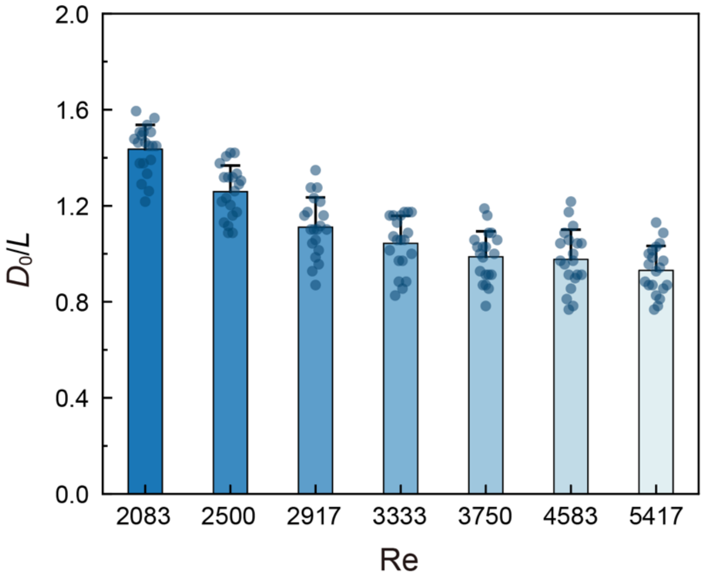

Supplementary Fig. 23. The ratio D_0/L of bubbles released in flow tests under varying Reynolds numbers Re. Error bars represent standard deviation calculated from 20 independent measurements.

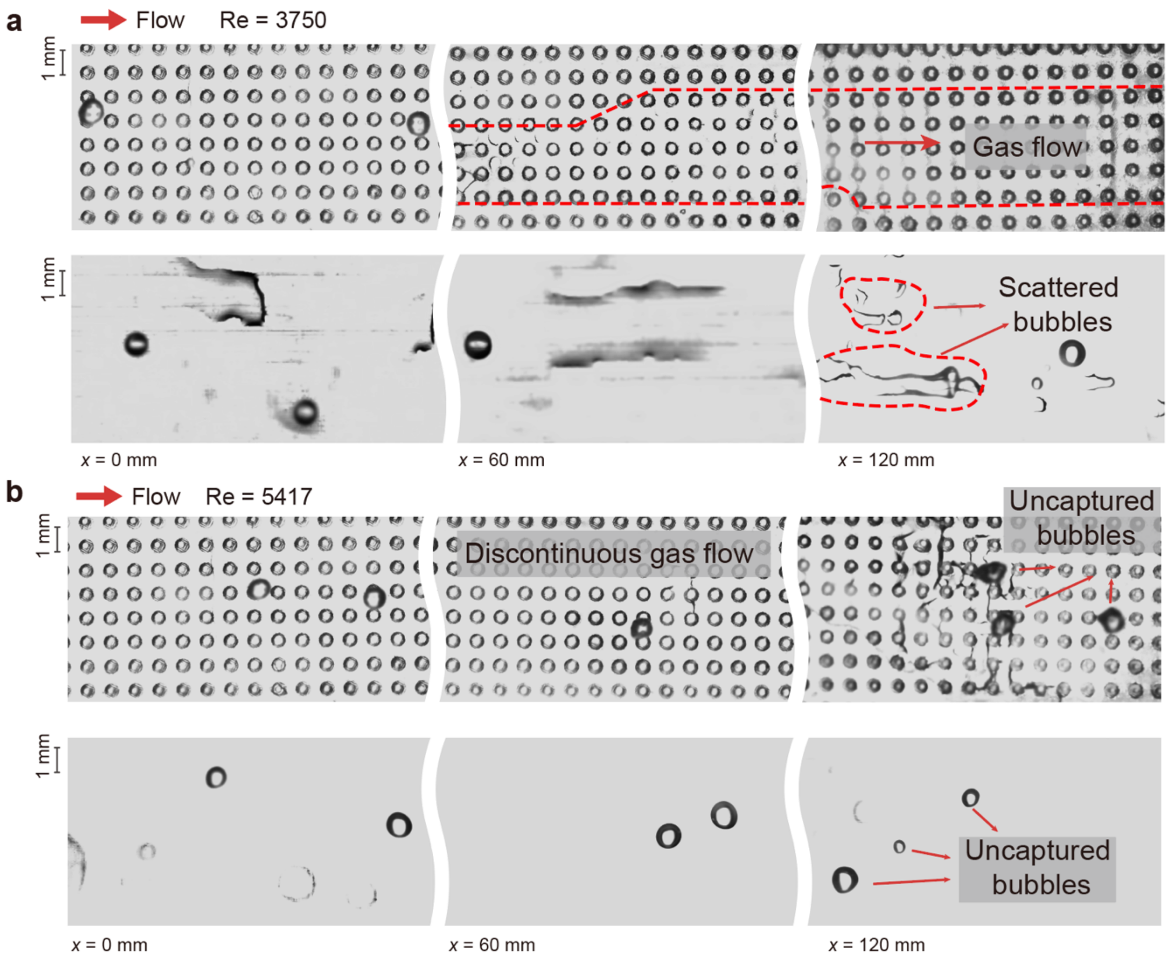

Supplementary Fig. 24. Bubble capture behaviors of MA surface and FH surface at Re of 3750 and 5417. Image sequences of capture behaviors on MA and FH surfaces along different surface locations at **a**, Re of 3750 and **b**, Re of 5417. Results show that more bubbles flow past the FH surface than that on the MA surface at Re = 3750. However, when the Re increases to 5417, the gas layer on the MA surface is destroyed with decreased capture efficiency, evidenced by the growing number of uncaptured bubbles.

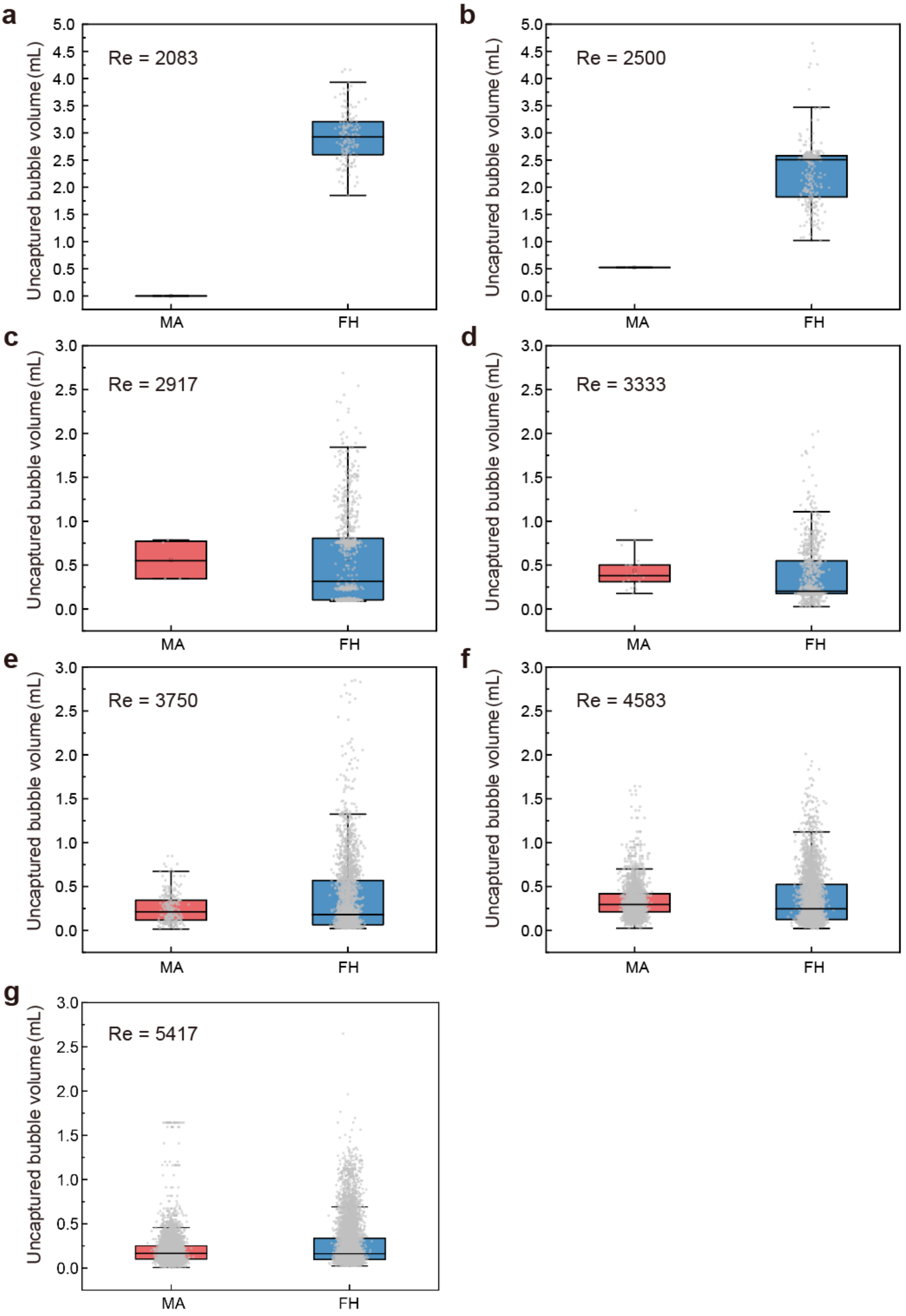

Supplementary Fig. 25. Volume distribution of uncaptured bubbles on the MA surface and FH surface at Re from 2083 to 5417. The boxes span from the 25th to the 75th percentile

of data. The horizontal black lines inside the boxes indicate the median value. The vertical black lines extend to 1.5 times the interquartile range of data from the boxes.

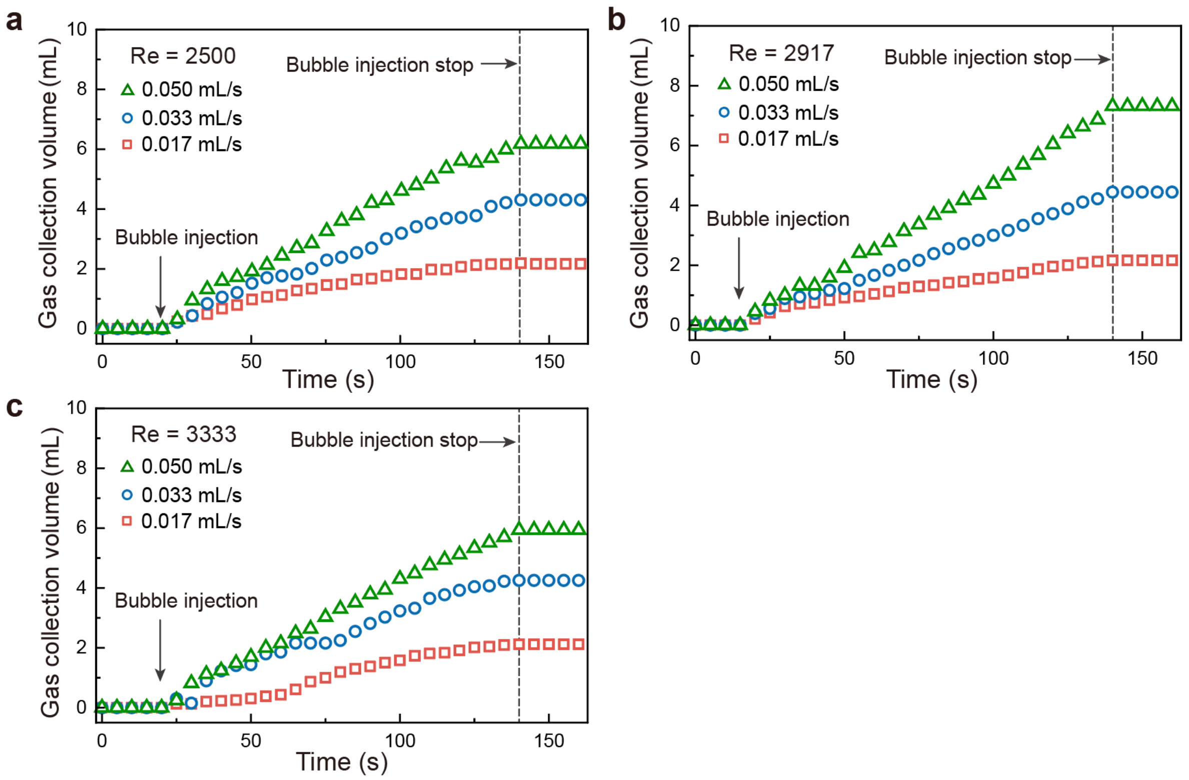

Supplementary Fig. 26. The collection gas volume under varying bubble injection rates and Reynolds numbers as a function of time. At Re numbers from 2500 to 3333, the MA surface maintains a bubble capture efficiency over 99% (Fig. 4g). Similar to the gas collection behavior at of 2083 (Fig. 4h), the collection volume curves over time all present nearly straight lines at all three injection rates at Re = 2500, 2917 and 3333, and the gas collection rates (indicated by the slopes of the curves) on MA surfaces are aligned with the injection rates, demonstrating the continuous loss-free gas transport.

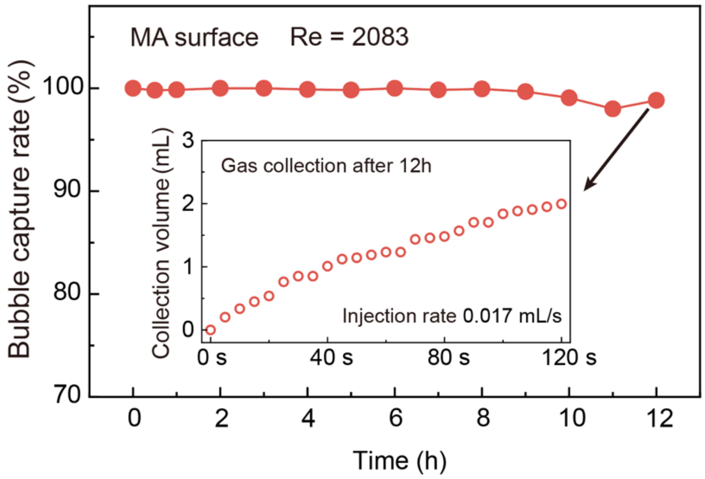

Supplementary Fig. 27. The evolution of bubble capture rate during 12 hours on the MA surface. The gas injection rate is 0.017 mL/s and the Reynolds number Re is 2083. A stable high efficiency is observed with all capture rates over 98%. The inset shows the collection volume as a function of time in 120 seconds after the 12-hour capture process.

Supplementary Tables

Supplementary Table 1. Summary of bubble capture time in previous work

Classification	Reference number in the main text	Materials	Structure Characteristic Size* (mm)	Bubble Diameter (mm)	Bubble Releasing Distance (mm)	Bubble Approaching Velocity (m/s)	Unified Capture Time (t_c, ms)
Micro-nano surfaces	[13]	Rough porous microstructures + deposited nanoparticles	0.0440	1.8	2.5	0.14	2.0–5.0
			0.0305				2.0–22.8
			0.0163				2.0–41.3
			0.0126				2.0–42.5
	[29]	Micro-agglomerates + sprayed superamphiphobic silica nanoparticles	0.0500	~1.9	0.28–73.7	~0.04–0.25	1.0–10.0
	[30]	Etched micro-nano textured silicon substrate + chemical modification	0.0150	~2.6	3.0	~0.16#	4.4
	[16]	Microporous membrane + hydrophobic nanoparticle coating	0.005	2.6	2.4	0.20	4.0
Microstructured surfaces (Inherently hydrophobic or with chemical modifications)	[36]	Rough PTFE substrate	0.0050	1.5	> 4.8	0.33	115.0 ± 17.0
	[37]	Rough Teflon substrate	0.0500	1.5	300	0.37	3.0
			0.0010				84.0
	[35]	Rough Teflon substrate	0.0050	1.5	250	0.35	105.0 ± 4.0
			0.0600				37.0 ± 3.6
			0.1000				2.0 ± 0.5
	[21]	Micropillars/microprotrusions + submicro features + silane modification	0.0100	~2.2	25	0.35	145.0
			0.0500	~2.0			50.0
			0.1680	~2.0			5.7
Nanostructured surfaces	[38]	PMMA nanofiber mat	0.0006	~2.3	3.6	0.16	203
	[26]	Silicon substrate + nanoparticle coating	0.0001	2.0	3.0-10.0	0.2–0.3	3.1–146.2

* Structure Characteristic Size S_c is the maximum characteristic structural size on each surface.

~ denotes extracted data from plots or videos of the literature, as these data are not directly provided.

The approaching velocity is estimated from bubble size and releasing distance.

Supplementary Table 2. Summary of capture time definitions and detection methods

Reference number in the main text	Capture Time Definition	Detection Method
[21]	The time from the initial deceleration from terminal velocity to the point at which the surface captures the bubble.	The velocity method#
[13]	The interval from the minimum decelerated velocity to the sharp increase in velocity detected.	
[29]	The time between contact (determined by the local minimum of the velocity–time graph) of the bubble with the surface and its rupturing (determined by the local maximum of the velocity–time graph).	
[16]	The instance of bubble detachment from the tip of the syringe needle to a sudden decrease in the bubble contact angle.	The visual method##
[36]	*The time of the TPC formation is the time span from the moment of the first collision to the three-phase contact line formation.	
[37]	*The time of attachment, i.e., the time period from a moment of the first collision to the TPC formation.	
[35]	*The time of the TPC formation is the time interval from the moment of the bubble first collision with the surface until the TPC perimeter assuring the bubble attachment is formed.	
[30]	*The time that the air bubble just contacted the lotus leaf surface before deformation to this complete spreading-out process.	

* Although different terms are used, these definitions of capture time are essentially the same as ours.

# The velocity method defines the start time when the bubble velocity begins to decrease or reaches zero, and the end time when the bubble experiences a sudden change in velocity due to capture. While this method is relatively automated and allows for quantitative detection, it can lead to inaccuracies. This is because the bubble may not exhibit a noticeable shift in the center of mass at the moment of rupture. Additionally, on the MA surface, the bubble continues to ascend even after it contacts the cone tip, and its velocity does not decrease to zero. Therefore, it is also difficult to accurately determine the starting time based on velocity changes.

The visual method generally identifies the start time when the bubble first touches the surface and the end time when the three-phase contact line forms (typically marked by a sudden decrease in the bubble contact angle). It is more accurate than the velocity method because the change in velocity may lag behind the bubble rupture event. While this method may be subject to some error due to limited spatial resolution, the error is less than 1 ms. Therefore, we find that the visual method is more accurate and reliable for our study.

Supplementary Notes

Note 1. The force balance model of the bubble approaching a surface

The force balance model is established to determine the motion of the rising bubble, where buoyancy force F_B, drag force F_D, added mass force F_A, and film drainage force F_F are considered in this process. The disjoining pressure is negligible here since the film thickness exceeds the effective range from the nanostructure. Due to the negligible density of gases relative to liquids, assuming the mass of bubble is zero[1], the equilibrium of the four forces is represented as

$$F_{\mathrm{B}} + F_{\mathrm{D}} + F_{\mathrm{A}} + F_{\mathrm{F}} = ma \approx 0. \tag{S1}$$

The forces F_B, F_D, F_A, and F_F are defined as follows:

$$\begin{cases} F_{\mathrm{B}} = -\frac{4}{3}\pi\rho g R_0^3 \\ F_{\mathrm{D}} = \frac{\pi}{4} C_{\mathrm{D}} Re\mu R_0 V \\ F_{\mathrm{A}} = \frac{4}{3}\pi\rho R_0^3 C_{\mathrm{m}} \frac{\mathrm{d}V}{\mathrm{d}t} - \frac{2}{3}\pi\rho R_0^3 \frac{\mathrm{d}C_{\mathrm{m}}}{\mathrm{d}h} V^2 \\ F_{\mathrm{F}} = \int_0^{r_{\mathrm{m}}} 2\pi r P_{\mathrm{f}}(r)\,\mathrm{d}r \end{cases}, \tag{S2}$$

where R_0 is the initial radius of bubble, h is the film thickness, g is the gravitational constant, ρ is the liquid density, μ is the viscosity, V is the bubble velocity, and $\mathrm{Re} = 2\rho|V|R_0/\mu$ is the instantaneous Reynolds number. $P_f(r)$ represents the film pressure that originated during the bubbles approaching. $r_m = 1.2R_0$ is the radial distance where the film pressure P_f has reduced to 0[2]. C_D is the drag coefficient predicted by Loth's theory[3]. C_m denotes the added mass coefficient which is assumed to be 0.5 here. Substitute Eq. S2 into Eq. S1 to obtain the force balance equation used in this model:

$$\frac{4}{3}\pi\rho R_0^3 C_{\mathrm{m}} \frac{\mathrm{d}V}{\mathrm{d}t} = \frac{4}{3}\pi\rho g R_0^3 - \frac{\pi}{4} C_{\mathrm{D}} Re\mu R_0 V + \frac{2}{3}\pi\rho R_0^3 \frac{\mathrm{d}C_{\mathrm{m}}}{\mathrm{d}h} V^2 - \int_0^{r_{\mathrm{m}}} 2\pi r P_{\mathrm{f}}(r)\,\mathrm{d}r. \tag{S3}$$

P_f in Eq. S3 can be simplified in average using flat film model (*45*), and explicitly given as

$$\bar{P}_{\mathrm{f}} = \frac{2\rho g D_0}{3}\left(1 - C_{\mathrm{m}} \frac{\mathrm{d}V}{\mathrm{d}t}/g\right)\left(\frac{D_0}{D_{\mathrm{b}}}\right)^2, \tag{S4}$$

which neglects the velocity-related terms since the bubble velocities become smaller than $(D_0 g)^{1/2}$ when bubbles close enough to the surfaces[4], where D_0 is the initial bubble diameter. For the estimation of C_m here, while the bubble deformation differs significantly among surfaces with varying b/a, the nominal 0.5 value is replaced by $C_m = \psi/(2-\psi)$, and $\psi = \frac{2\chi^2}{\chi^2-1}\left(1 - \frac{1}{\sqrt{\chi^2-1}}\cos^{-1}\left(\frac{1}{\chi}\right)\right)$, where $\chi = D_h/D_v$ is the transverse and longitudinal ratio of bubble. Eq. S4 is only used for the film pressure estimation in Fig. 3h. We estimate the maximum average film pressure $\bar{P}_m$ based on Eq. S4 during the bubble contact by using the maximum deceleration $(\mathrm{d}V/\mathrm{d}t)_m$, and the contemporaneous χ and the bubble basal diameter D_b from the experimental results.

Note 2. Theoretical modeling of the LGI deformation

The dynamics of the film is described based on the Stokes-Reynolds-Young-Laplace equation, coupling with the force balance model in Note 1. The shape of the liquid-gas interface z(r, t) and the bubble surface $z_b(r, t)$ is defined by the Young-Laplace equation, which can be described as:

$$\frac{\sigma}{r}\frac{\partial}{\partial r}\left(r\frac{\partial z}{\partial r}\right) = \rho g z - P_{\mathrm{f}}, \tag{S5}$$

$$\frac{\sigma}{r}\frac{\partial}{\partial r}\left(r\frac{\partial z_{\mathrm{b}}}{\partial r}\right) = -\frac{4\sigma}{D_0} + P_{\mathrm{f}}. \tag{S6}$$

The film pressure P_{f} is derived by subtracting Eq. S5 from Eq. S6:

$$P_{\mathrm{f}} = \frac{2\sigma}{D_0} + \frac{\rho g z}{2} - \frac{\sigma}{2r}\frac{\partial}{\partial r}\left(r\frac{\partial h}{\partial r}\right), \tag{S7}$$

where σ is the liquid-gas interfacial tension, and the film thickness $h(r, t) = z - z_b$. To describe the thinning of the film, the drainage rate is determined using lubrication theory[5]:

$$\frac{\partial h}{\partial t} = \frac{1}{3\mu r}\frac{\partial}{\partial r}\left(r h^3 \frac{\partial P_{\mathrm{f}}}{\partial r}\right). \tag{S8}$$

The corresponding prefactor of this equation is chosen to be 1/3 in our work. The boundary conditions guide our choice of the prefactor. In this case, the lower boundary of the liquid film is the bubble surface, which can be approximated as the slip boundary (no added medium in the water). This approximation is based on the fact that, in a pure water environment, the gas-liquid interface cannot withstand shear stress, particularly in the absence of contaminants or surfactants. However, it should be noted that clean bubble surfaces may transition to a no-slip conditions upon collision with a solid surface[5], suggesting that the slip boundary applicability may be constrained by specific conditions. Additionally, gas-liquid interface mobility at the thin-film level remains controversial: millisecond-scale bubble coalescence[6,7] has been speculated to be a result of interfacial mobility, yet direct observations continue to support the immobile boundary condition[8]. The precise description of boundary conditions in this context also requires further exploration.

The liquid film's upper boundary is the gas-liquid interface within the microstructure. Considering bubbles smaller than the microstructure spacing, it should be approximated as a free liquid surface or no-slip boundary[9].

By solving Eq. S7 and Eq. S8 simultaneously, the film drainage rate $\partial h/\partial t$ and the film thickness h are obtained and further determine the P_{f} at each timestep. The initial condition for the film thickness assumes $h(r, 0) = h_0 + r^2/2R_0$, where h_0 is set to 20 mm, the same as in the experiment. The initial condition $P_{\mathrm{f}}(r, 0)$ can be calculated through Eq. S7 with the initial condition $h(r, 0)$. The boundary condition of the drainage rate is defined separately on the MA and FH surfaces. For the MA surface, considering the pinning effect of the three-phase contact lines on microstructures, the drainage rate at $r = r_{\mathrm{m}}$ assumes equal to the motion velocity of the bubble, $\frac{\mathrm{d}h}{\mathrm{d}t}|_{r=r_{\mathrm{m}}} = V$. On the FH surface, the liquid-gas interface deformation rate should be subtracted from the drainage rate at the free boundary without microstructures, which can be written as $\frac{\mathrm{d}h}{\mathrm{d}t}|_{r=r_{\mathrm{m}}} = V - \frac{\partial z}{\partial t}$.

To calculate the liquid-gas interface profiles z, Eq. S5 and Eq. S7 are solved with the P_{f} obtained as above. The boundary condition z($r = \pm L/2$) is also determined separately in the cases with and without microstructures. When microstructures exist, z($r = \pm L/2$) is set as zero

due to the pinning effect of the three-phase contact lines. On the surface without microstructure, the film pressure at $r = r_m$ is assumed to be zero, thus Eq. S5 can be solved using a second-order improved Bessel function and further obtain that $z(r_m, t) = \frac{F_F(t)}{2\pi\sigma}[K_0(r_m/\sqrt{\frac{\sigma}{\rho g}}) - [K_0(L/\sqrt{\frac{4\sigma}{\rho g}})]$, where K_0 is modified Bessel function of the second kind of order zero.

Note 3. SPH simulation of bubble capture on MA surfaces

The numerical simulations are performed based on an SPH solver for multiphase flow enhanced by MUSCL scheme[10]. The simplified Navier-Stokes equations with surface tension force and without viscous force in momentum equation are written as

$$\begin{cases} \dfrac{\mathrm{d}\rho}{\mathrm{d}t} = -\rho\nabla\cdot\boldsymbol{v} \\ \rho\dfrac{\mathrm{d}\boldsymbol{v}}{\mathrm{d}t} = -\nabla p + \rho\boldsymbol{g} + \boldsymbol{f^{st}}, \\ \rho\dfrac{\mathrm{d}e}{\mathrm{d}t} = -p\nabla\cdot\boldsymbol{v} \end{cases} \tag{S9}$$

where $\boldsymbol{v}$ is the velocity, e is the internal energy per unit mass, p is the pressure, $\boldsymbol{g}$ is the acceleration of gravity, $\boldsymbol{f^{st}}$ is the surface tension force. The non-conservative differential formulation is adopted here to derive the Lagrangian particle description in SPH method. Interface mobility is crucial for the liquid film dynamics[11,12], and here we treat all interfaces (including gas-liquid and solid-liquid interfaces) as mobile interfaces with free slip, due to the dominant role of surface tension. The behavior of an individual bubble in a stagnant fluid is dominated by two non-dimensional parameters, namely the Eötvös number Eö = $\Delta\rho g s^2/\sigma$ and the Morton number Mo = $\Delta\rho g \mu_c^4/\rho_c^2\sigma^3$, where $\Delta\rho$ is the absolute value of the density difference between the two phases, s is the characteristic length (bubble radius in this study). ρ_c and μ_c are the mass density and viscosity coefficient of the surrounding fluid, respectively. Eö describes the ratio of gravitational and capillary forces, while Mo describes the ratio of viscous and capillary forces. Considering the physical parameters of water and air at room temperature, we have Mo $\approx 3.7 \times 10^{-11} \ll$ Eö $\approx 0.06 \ll 1$, indicating the shape of multiphase interfaces is mainly controlled by surface tension.

To describe the compressibility of the gas and liquid phases, the Tammann equation $p = (\omega - 1)\rho e - \omega B$ is utilized for both air and water phases, where ω is the specific heat ratio, B is a material constant, and $B = 0$ represents the ideal gas state. For air, we set ρ_a^0 = 1.29 kg/m^3, ω_a = 1.4, B_a = 0 Pa; for water, ρ_w^0 = 1000 kg/m^3, ω_w = 7, $B_w = 4.485 \times 10^4$ Pa, ρ^0 represents the reference mass density. Eq. S9 is discretized by Riemann SPH solver, which can eliminate pressure oscillations and free from parameter tuning. To describe the macroscopic surface tension effect, the continuum surface force (CSF) model[13] is embedded in our SPH framework by converting the surface tension force into a body force, written as $f_i^{st} = -\sigma\kappa_i\boldsymbol{n}_i$ for particle i, where κ_i is the local curvature, $\boldsymbol{n}_i$ is the unit outer normal vector on the interface. A correction force parallel to the wall surface associated with the advancing angle (162°, Supplementary Fig. 2) applied near the TPL to realize the dynamic control of the contact angle. The scale of TPL region is defined by the radius of kernel function. In addition, dummy particle boundary conditions[14] are applied to the solid boundary conditions of SPH. We induce a particle regeneration technique[15] that is periodically triggered to keep the liquid-gas interface distinct. A predictor-corrector scheme is used for time integration.

Note 4. Scaling law of energy transition

Regarding the scaling law in our work, we theoretically postulate that a fraction of kinetic energy at terminal velocity V_{t} is transmuted into the bubble's surface energy during the bubble's approach, which can be written as $\alpha E_{\mathrm{k}} = E_{\sigma}$, where $E_{\mathrm{k}} \sim \rho^{*} D_0^3 V_{\mathrm{t}}^2$ and $E_{\sigma} \sim \gamma \eta_{\mathrm{terminal}}^2$. Here, α denotes the energy transfer efficiency, ranging from 0 to 1, η_{terminal} is the ultimate deformation of the bubble contacting the surface, and $\rho^{*} = C_{\mathrm{m}} \rho$ is the density considering total inertia in the motion of the bubble, where ρ is the liquid density and C_{m} is the added mass coefficient. Accordingly, substituting E_{k} and E_{σ} into the formula, we derive that $\eta_{\mathrm{terminal}}/D_0 \sim (\alpha \mathrm{We}^{*})^{1/2}$, with $\mathrm{We}^{*} = \rho^{*} D_0 V_{\mathrm{t}}^2/\gamma$.

Note 5. Derivation of the critical pressure ΔP_{cr}

For the microconical surface, the three-phase contact lines pinned on microstructures provide a force that can resist pressure, which can be expressed as

$$F_\sigma = 2\pi a_c \sigma \cos\theta_E , \tag{S10}$$

where a_c is the cross-section radius of the microstructure where the three-phase contact line forms. Considering that the gas layer will move under drainage pressure but may also cause film break with the ascending bubble, we simplify $a_c = a/2$, which represents the average position of the three-phase line as it moves, to predict the rapid capture of bubbles[16]. $\theta_E = \pi + \alpha - \theta_{adv}$, is the angle between the interfacial tension force F_σ and the vertical axis, where α equals to half the apex angle of the cone, and $\theta_{adv} = 162°$ is the advancing angle of the surface. If θ_E exceeds $\pi/2$ when the geometric parameter b/a of cones is too small, F_σ is considered as zero because the surface is unable to form gas layers between the microstructures. The critical pressure difference can be calculated by

$$\Delta P_{cr} = \frac{F_\sigma}{A} = \frac{2\pi a_c \sigma \cos\theta_E}{L^2 - \pi a_c^2} . \tag{S11}$$

Based on this equation, the general Laplace expression can be evolved as

$$\Delta P_{cr} = \frac{\sigma \cos\theta_E}{R_c} , \tag{S12}$$

where $R_c = A/C$ is the capillary radius[16] of meniscus' spatial curvature with the projected area of the liquid-gas interface $A = L^2 - \pi a_c^2$, and the length of the three-phase contact line $C = 2\pi a_c$ (as illustrated in Supplementary Fig. 17).

Notably, although surface nanostructures can affect the estimation of contact line length, under Cassie wetting conditions, this error is only about 1% and can therefore be neglected. We have considered the influence of nanostructures on surface hydrophobicity by introducing the advancing contact angle (θ_{adv}). The θ_{adv} is a parameter that comprehensively reflects both the surface chemical properties and morphology. The impact of nanostructures on surface hydrophobicity and contact line behavior is indirectly reflected through the effect of θ_{adv} on the θ_E value in Equation S12.

Supplementary References

1. Manica R., Klaseboer E., Chan D. Y. C. Force balance model for bubble rise, impact, and bounce from solid surfaces. *Langmuir* **31**, 6763–6772 (2015).
2. Manica R., Klaseboer E., Chan D. Y. C. The impact and bounce of air bubbles at a flat fluid interface. *Soft Matter* **12**, 3271–3282 (2016).
3. Loth E. Quasi-steady shape and drag of deformable bubbles and drops. *Int. J. Multiphase Flow* **34**, 523–546 (2008).
4. Rapoport L., Emmerich T., Varanasi K. K. Capturing bubbles and preventing foam using aerophilic surfaces. *Adv. Mater. Interfaces* **7**, 1901599 (2020).
5. Liu B., Manica R., Liu Q. X., Klaseboer E., Xu Z. H., Xie G. Y. Coalescence of bubbles with mobile interfaces in water. *Phys. Rev. Lett.* **122**, 194501 (2019).
6. Del Castillo L. A., Ohnishi S., Horn R. G. Inhibition of bubble coalescence: Effects of salt concentration and speed of approach. *J. Colloid Interface Sci.* **356**, 316–324 (2011).
7. Vakarelski I. U., Kamoliddinov F., Thoroddsen S. T. Why Bubbles Coalesce Faster than Droplets: The Effects of Interface Mobility and Surface Charge. *Langmuir* **40**, 11340–11351 (2024).
8. Hendrix M. H. W., Manica R., Klaseboer E., Chan D. Y. C., Ohl C. D. Spatiotemporal evolution of thin liquid films during impact of water bubbles on glass on a micrometer to nanometer scale. *Phys. Rev. Lett.* **108**, 247803 (2012).
9. Emery T. S., Kandlikar S. G. Modeling bubble collisions at liquid-liquid and compound interfaces. *Langmuir* **35**, 8294–8307 (2019).
10. Li M. K., Zhang A. M., Peng Y. X., Ming F. R. An improved model for compressible multiphase flows based on Smoothed Particle Hydrodynamics with enhanced particle regeneration technique. *J. Comput. Phys.* **458**, 111106 (2022).
11. Vakarelski I. U., Langley K. R., Yang F., Thoroddsen S. T. Interferometry and simulation of the thin liquid film between a free-rising bubble and a glass substrate. *Langmuir* **38**, 2363–2371 (2022).
12. Vakarelski I. U., Yang F., Thoroddsen S. T. Free-rising bubbles bounce more strongly from mobile than from immobile water-air interfaces. *Langmuir* **36**, 5908–5918 (2020).
13. Huber M., *et al.* On the physically based modeling of surface tension and moving contact lines with dynamic contact angles on the continuum scale. *J. Comput. Phys.* **310**, 459–477 (2016).
14. Zhang C., Zhu Y. J., Lyu X. X., Hu X. Y. An efficient and generalized solid boundary condition for SPH : Applications to multi-phase flow and fluid-structure interaction. *Eur. J. Mech. B-Fluid* **94**, 276–292 (2022).
15. Peng Y. X., Zhang A. M., Ming F. R. Particle regeneration technique for Smoothed Particle Hydrodynamics in simulation of compressible multiphase flows. *Comput. Methods Appl. Mech. Eng.* **376**, 113653 (2021).
16. Zhao Y. P., *et al.* Recoverable underwater superhydrophobicity from a fully wetted state via dynamic air spreading. *iScience* **24**, 103427 (2021).
