# Supplementary material for: Understanding ultrafast free-rising bubble capturing on nano/micro-structured super-aerophilic surfaces
Source: Nat Commun. 2025 Apr 17;16:3682. doi: 10.1038/s41467-025-59049-x (PMC12006317; doi:10.1038/s41467-025-59049-x)
Supplement: Supplementary file 2 — Description of Additional Supplementary Files [file 41467_2025_59049_MOESM2_ESM.docx]

Description of Additional Supplementary Files

File Name: Supplementary Movie 1

Description: Ultrafast bubble capture on Salvinia leaves.

File Name: Supplementary Movie 2

Description: Comparison of bubble capture on the MA and the FH surfaces.

File Name: Supplementary Movie 3

Description: Evolution of the gas layer profile under fluorescent reflection on the MA and the FH surfaces.

File Name: Supplementary Movie 4

Description: Bubble capture behavior in flow environments on the MA and the FH surfaces.

File Name: Supplementary Movie 5

Description: Gas collection in a flow environment through the MA surface under varying injection rates.

File Name: Supplementary Movie 6

Description: Bubble collection in 12 hours on the MA surface in a shear flow environment.
